# EFPT Exchange programme - Feedback results from 2012 - 2022

**DOI:** 10.1192/j.eurpsy.2024.235

**Published:** 2024-08-27

**Authors:** F. H. Kraxner

**Affiliations:** Department of Psychiatry and Psychotherapy, Hospital of Affoltern, Affoltern am Albis, Switzerland

## Abstract

**Introduction:**

Differences in psychiatry training vary substantially across Europe. Such organisations as the European Federation of Psychiatry Trainees (EFPT), the European College of Neuropsychopharmacology, and the European Psychiatry Association, have committed to offer international experiences based on the premise that it could foster international collaboration, aid early career professionals to progress professionally and spark discussion regarding different practices across Europe. To date, there are no studies that focus exclusively on the exchange experience in mental health professionals

**Objectives:**

I present the synthesis of the ten years answers from 2012 to 2022 to the post-exchange online evaluation form, which trainees had to fill in in order to receive an attendance certificate.

**Methods:**

The present study analysed the answers of 202 psychiatry trainees or recent graduates who took part in the EFPT exchange program during 2012 and 2022 and filled in the internet-based evaluation form. The inclusion criteria were currently in training or recently finished training as a psychiatrist in Europe and filling in the questionnaire. The exclusion criterion was participation in the EFPT exchange program for the second or subsequent time.

All trainees were systemically asked to complete the online evaluation form after the exchange period. The form includes sociodemographic, training in host country-related, and exchange experience-related questions. Experience measures were evaluated using the 4-point Likert scale. Data was anonymized before the analysis. The study followed the principles of the Declaration of Helsinki.

**Results:**

The majority of participants were females in the second half of their training. The average age was 29 years. The largest number of applicants were from Turkey, whereas the United Kingdom hosted the most participants. One-third of the participants had previous international exchange experience. Most trainees were exposed to both outpatient and inpatient treatment settings and were involved in educational or research activities. 96.7% of participants indicated that they were satisfied or very satisfied with the experience, 95.6% said that the exchange was useful or very useful, and 98.9% were likely or very likely to recommend exchange to colleagues.

**Conclusions:**

To my knowledge, this study is the first to assess the experience of psychiatry trainees who went on exchange during their professional training. Vast majority of trainees were satisfied with their exchange, thought it would be useful for their clinical practice and would recommend it to their colleagues. These findings are in line with other studies that examined medical exchange experiences .

**Disclosure of Interest:**

None Declared

